# Covariates Relating to Implant Failure and Marginal Bone Loss of a Novel Triangular Neck-Implant Placed by Post-Graduate Students: A 1-Year Prospective Cohort Study

**DOI:** 10.3390/ma15061987

**Published:** 2022-03-08

**Authors:** Maria Giralt-Hernando, Gian Maria Ragucci, Oriol Cantó-Naves, Adaia Valls-Ontañón, Federico Hernández-Alfaro

**Affiliations:** 1 Department of Oral and Maxillofacial Surgery, Universitat Internacional de Catalunya (UIC), Sant Cugat del Vallés, 08195 Barcelona, Spain; gian1@uic.es (G.M.R.); avalls@uic.es (A.V.-O.); h.alfaro@uic.es (F.H.-A.); 2 Department of Restorative Dentistry, School of Dentistry, Universitat Internacional de Catalunya (UIC), Sant Cugat del Vallés, 08195 Barcelona, Spain; oriolcanto@uic.es; 3 Institute of Maxillofacial Surgery, Teknon Medical Center, Edifici Vilana desptx 185, 08022 Barcelona, Spain

**Keywords:** marginal bone loss, dental implants, peri-implantitis, keratinized tissue, implant neck, success rate, failure rate

## Abstract

(1) Background: Most of the clinical literature dealing with dental implants has been issued by experienced teams working either in university settings or in private practice. The purpose of this study was to identify contributing covariates to implant failure and marginal bone loss (MBL) at the 1-year follow-up of a novel triangular-neck implant design when placed by inexperienced post-graduate students. (2) Methods: A prospective cohort study was conducted on study participants eligible for implant placement at the UIC (International University of Catalonia), Barcelona, Spain. Implant failure rate and contributors to implant failure and MBL were investigated among 24 implant and patient variables. (3) Results: One hundred and twenty implants (V3, MIS) were placed and rehabilitated by the students. The mean insertion torque was 37.1 Ncm. Survival and success rates were 97.5% and 96.7%, respectively. Implants placed in patients with smoking habits displayed a tendency of higher failure risk (OR = 5.31, *p* = 0.17) when compared to non-smokers. The mean (SD) MBL was 0.51 (0.44) mm. Gender significantly affected the MBL (*p* = 0.020). Bleeding on probing (BoP) on the buccal sites proved to be a good predictor of proximal MBL (*p* = 0.030). (4) Conclusions: The survival and success rates of the V3 triangular-neck implant placed by inexperienced post-graduate students at the 1-year follow-up were high and similar to the ones published in the literature by experienced teams on other implants.

## 1. Introduction

Implant therapy for oral rehabilitation has been widely accepted as a standard treatment to rehabilitate partially and totally edentulous patients [1]. Long-term implant survival rates of 96.4% have been described in the literature [2]. However, most of the clinical literature dealing with dental implants has been issued by experienced teams working either in university settings or in private practices. Documentations of clinical outcomes in the hands of young inexperienced post-graduate students have been scarce [2]. 

Careful patient selection and treatment planning have been stressed [2]. Proper three-dimensional (3D) placement of the implant, patients’ soft tissue biotype, and thickness are factors that play a major role in achieving predictable outcomes [2]. Besides these important factors, the buccal wall should display a minimum thickness in order to avoid future complications [3]. 

Micro- and macro-features of implant design include a large range of variables; among them are the geometry and surface of the implant body and its neck [2,3,4,5,6,7,8,9]. Several papers have documented the implant neck as a critical factor involved in preservation of the marginal peri-implant bone [1,3,4,10,11]. To date, the most common implant collar design is the conventional circular one [1,10]; recently, however, a new conical triangular-neck design with three flat and one conical connection surfaces has been made available on the implant market (V3, MIS Implant Technologies, Bar Lev Industrial Park, Israel). This shape has been implemented with several objectives in mind. One was to reduce the stresses and strains that are exerted at the cortical bone during implant seating [12]; the second was to offer added room at the implant neck for thickening the buccal cortical bone lamella [4,5,6] under the motto less titanium, more bone, and third to speed-up the neoformation of the crestal bone [13]. When the implant is seated, the triangular neck design provides a space between the implant collar and the surrounding bone to be filled with blood; it eventually forms a blood clot, which later turns into bone [2,3,4,6,7,8,9]. 

All these above addressed issues are cardinal to prevent complications and reduce the occurrence of aesthetic and biological failures [3], but documentation of this novel triangular neck shape has been limited so far and was provided by experimented clinicians only [3,4,6,8,12]. Therefore, the purpose of this prospective cohort study was to evaluate, at the 1-year follow-up, the success and survival rates as well as the marginal bone loss (MBL) of the novel V3 triangular-neck implant design when placed and rehabilitated by young inexperienced post-graduate students. The null hypothesis of this prospective cohort study was that there is no difference at the one-year follow-up between the clinical performance of implant placement by post-graduate students of a new triangle implant design regarding MBL and implant failure.

## 2. Materials and Methods

### 2.1. Study Design

A prospective cohort study was designed at the Department of Oral and Maxillofacial Surgery of the Universitat Internacional de Catalunya (medicine campus Sant Cugat del Vallés, Barcelona, Spain) after approval by the Ethics Committee of the university (CIR-ECL-2015-06). Patients in need of implant therapy that received the V3 implant from January 2016 to January 2017 were part of this study. The neck of this implant is unique and innovative; its triangular design challenges the common cylindrical shape of the collar. The three resulting flat surfaces reduce the area of tight contact between the implant neck and the cortical bone of the alveolar ridge; final implant seating leaves a bony gap that varies between 0.1 to 0.5 mm according to the diameter of the implant. The coronal part of this tapered implant implements a twelve degrees internal conical connection and platform-switching. The cutting blades at the implant apex establish self-tapping properties; the flat cutting apex allows for final corono-apical adjustments. It is made of titanium grade 23, an alloy similar to titanium grade 5, but with reduced interstitial elements of oxygen and iron that increase the strength of the material. Its surface is sandblasted, and acid etched, and the Sa is 1.22 µm [7].

### 2.2. Patient Selection—Inclusion/Exclusion Criteria

All indications, single crown, fixed partial dentures, and full-arch rehabilitation were included. Inclusion criteria were the following: (a) patients older than 18 years in need of implant therapy, (b) good general/systemic health (ASA type I, II), (c) patients who committed to attend all visits of the study, (d) those that underwent or required a bone regeneration procedure, horizontal or vertical guided bone regeneration with or without resorbable membrane or block graft, (e) sinus lift, (f) adequate oral hygiene with FMPS (full mouth plaque score) < 15% before surgery, (g) absence of uncontrolled periodontal disease, (h) agreeing to sign an informed consent. 

Exclusion criteria were the following: (a) patients with a contributing medical history in which any surgery, disease, condition, or medication that might compromise the healing of soft and hard tissues (e.g., non-controlled diabetes), (b) liver function disorder, (c) immune system disease, (d) immunosuppressant drugs, (e) toxic habits other than smoking that might compromise or affect healing, (f) patients who have undergone chemotherapy or radiation treatment during the previous 5 years comprising the head and neck area, (g) corticosteroids therapy or any other medication that could influence postoperative healing and/or osseointegration, (h) bisphosphonate or Denosumab therapy (Prolia^®^), (i) inability or unwillingness to attend follow-up visits, (j) patients unwilling to sign an informed consent form.

### 2.3. Surgical Procedure

All of the surgical and prosthetic procedures were performed at the Clinic of Dentistry of the University (CUO) by 24 young 24–27-year-old post-graduate students that just completed their dental school curriculum. Before implant placement, the diagnostic protocol included a diagnostic wax-up in order to obtain a radiological guide. A cone-beam computed tomography (iCAT^®^, Imaging Science International, 2800 Crystal Drive, Hatfield, PA, USA) was taken in the target area with the respective radiographic guide to get 3D guidance for implant selection and 3D positioning. The drilling sequence was performed according to the recommendation of the manufacturer by using each drill, including the final drill, which is delivered with each implant as a disposable tool. With the help of the specific insertion device that displays 3 flat areas as well, the implants were placed with one of the flat sides parallel to the vestibular bone lamella. Depending on the maximum insertion torque (IT) recorded by the surgical motor (Implantmed W&H^®^), ≥ or < to 35 Ncm, either a healing abutment or a cover screw were placed. The 1-stage protocol was performed by placing a healing abutment and tissue approximation using single stitches; for the 2-stage protocol, a cover screw was placed, and primary flap closure was achieved over it. Patients received antibiotic (875/125 mg of Amoxicillin/Clavulanic acid, 3×/d for 7 days; in case of penicillin allergy, 300 mg of Clindamycin every 6 h for 7 days) and analgesic anti-inflammatory treatment (600 mg Ibuprofen 3×/d); rinsing with Chlorhexidine (0.12%) (Dentaid^®^ PerioAid 0.12%) was prescribed 2×/d for 2 weeks.

After 7 days, the patients were recalled for suture removal and then again after one month. After 3 months of healing in the mandible and maxilla, osseointegration was checked clinically and radiographically. The prosthetic phase (T1) was initiated; the partial or complete ceramo-metallic prostheses were seated on multi-unit abutment (MUA) while the single ceramo-metallic crowns were cemented to a titanium base. The MUA was no more disconnected from the implant neck according to the one-abutment one-time principle; otherwise, the implant-abutment junction (IAJ) was disconnected over 3–5 times.

### 2.4. Study Variables and Measurements

Demographic parameters of the participants, such as age, sex, smoking status, and medical history, were recorded. The recorded and assessed variables regarding the characteristics of the participant, the implant, the surgical site, and the prosthetic outcomes are shown in Table 1. Success rates were assessed according to the criteria of Albrektsson and Zarb [14] and later modified by Buser et al. [15].

#### 2.4.1. Radiographic Assessment

**Figure 1:**
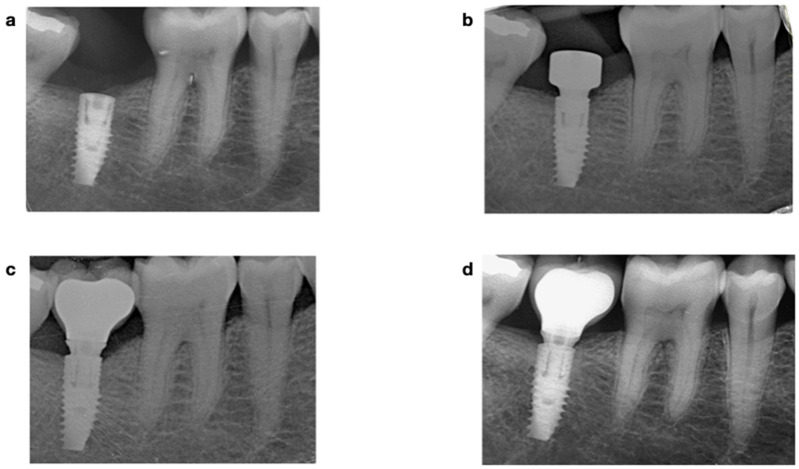
Periapical radiograph-parallel technique for MBL analysis at the 1-year follow-up. (**a**) 2-stage implant placement; (**b**) 2-stage surgery 4 months after implant placement; (**c**) CAD-CAM metallo-ceramic crown cemented to a 0.5 mm anti-rotatory Ti-base abutment at the 6-month follow-up; and (**d**) at the 1-year follow-up.

Periapical radiographs of each implant were acquired with an intraoral dental film using a plastic index according to the parallel technique immediately after implant placement, at prosthesis delivery, and at the 1-year follow-up. Measurements were performed with the ImageJ software (NIH^®^, Bethesda, Rockville, MD, USA); internal calibration was provided by the diameter of the implant neck. At each time point, the distance from the implant shoulder to the first bone-implant contact was measured on the mesial and distal sites. The difference between baseline and milestone served to calculate the MBL on each side. Subsequently, the mean value of the two measurements was calculated for each implant (Figure 1). 

#### 2.4.2. Clinical Assessment

Peri-implant clinical parameters were assessed at four sites (mesial, buccal and distal, and lingual) with the use of a periodontal probe (UNC 15, Hu-Friedy^®^, Chicago, IL, USA) in the following way: (a) probing depth (PD) in millimeters was measured from the peri-implant mucosal margin to the bottom of the peri-implant sulcus; (b) bleeding on probing (BoP) was determined as presence or absence of bleeding 15 s after gentle probing; (c) keratinized tissue (KT) width in millimeters was measured with a periodontal probe at the mid-buccal aspect of the implant from the free gingival margin to the muco-gingival junction. Furthermore, the KT measurements were categorized in two groups, group 1 when KT ≥ 2 mm and group 2 when KT < 2 mm; (d) depth of implant placement measured on the proximal sides. In addition, other variables such as implant location (maxilla or mandible, anterior or posterior) and type of the implant-supported fixed dental prosthesis single crown (SC) or fixed partial denture (FPD) were also recorded.

### 2.5. Statistical Methods

Two investigators (MGH and GMR) independently evaluated the clinical parameters at the 1-year follow-up. If any differences arose, the scores were then discussed with a senior researcher (FHA). Clinical and radiographic examinations were performed following the same procedures at baseline (T0), prosthesis delivery (T1), and 1-year follow-up (T2). Descriptive data of the parameters analyzed at participant and implant levels were the following: mean (standard deviation), minimum, maximum, and median for the continuous variables, absolute frequencies, and percentages for the categorical ones. The probability of failure at the implant level based on each of the independent factors and covariates was determined with a simple binary logistic regression with GEE models obtaining unadjusted odds ratios (OR) as a function of the factors of profile. The relationship between MBL and the independent variables was assessed using a simple linear regression estimated with GEE models and the Chi-square statistic test of Wald. Significance was set at *p* < 0.05. The sample size calculation was as follows: a sample of *n* = 100 implants reached a power of 77.4% to detect a large effect size of d = 0.8 in the average difference of MBL between 2 statistically significant outcomes, assuming a level of confidence of 95% (CI).

## 3. Results

A total of 120 V3 implants were placed by 24 students in 47 participants, 22 females (46.8%) and 25 males (53.2%), mean age was 48.6 (10.2) years; 23.4% (*n* = 11) were smokers with less than 10 cigarettes/day, 44.7% had a previous history of periodontal disease that was under control at the time of implant treatment and 14.9% suffered from diabetes Mellitus type II. Implants placed in the mandible and the maxilla were 45% (*n* = 54) and 55% (*n* = 66), respectively; the majority of the implants (*n* = 79, 65.8%) were located in the posterior zone; 16.8% of the implant sites required bone grafting. 

Implant diameters were 3.3–5 mm, and implant lengths were 8–13 mm; most implants were Ø 3.9 and Ø 4.3 mm, and lengths were 10 and 11.5 mm. The IT varied from 20 to 60 Ncm; the mean and median were 37.1 ± 10.6 and 40 Ncm, respectively. Table 2a,b displays the IT according to the diameter and length of the implants; the differences were not statistically significant. When assessing the sample according to bone quality, the mean and median of type 2 and 3 were 39.5 ± 10.1 and 40 Ncm vs. 33.6 ± 10.1 and 35 Ncm, respectively, and the difference was statistically significant (*p* = 0.018). The number of implants placed in bone types 1 and 4 was limited; therefore, their mean and median were not calculated. The mean and median IT of the anterior and posterior sites were 36.43 ± 11.1 Ncm and 40 Ncm vs. 37.8 ± 10.2 Ncm, median 40 Ncm, respectively; the difference was not statistically significant (*p* = 0.28). Finally, the mean and median IT of the implants inserted in the posterior maxilla were 39.0 ± 10.4 and 40 Ncm, respectively.

Late implant loading (≥3 months after implant placement) was assessed for the majority of the implants (92.4%). Single crowns and full-arch rehabilitations were 49.6% and 10.3%, respectively. The 99.1% of all rehabilitations were screw-retained.

### 3.1. Implant Survival

Three implants failed before prosthetic loading, all in the posterior area of distinct patients—two in the mandible and one in the maxilla. The implant survival rate was 97.5% with 95% (CI 92.9–99.5%); 6.4% of the patients experienced a failure. Table 3 describes the association between failure and the different covariates at both the patient and implant levels. No variable was found to significantly affect the survival rate (Table 3). Nevertheless, a certain association was found for implants placed in patients with smoking habits (*p* = 0.172) in which implant failure was ×5 times higher (OR = 5.31) in smokers when compared to non-smokers (Table 3).

### 3.2. Marginal Bone Loss and Implant Success

**Figure 2:**
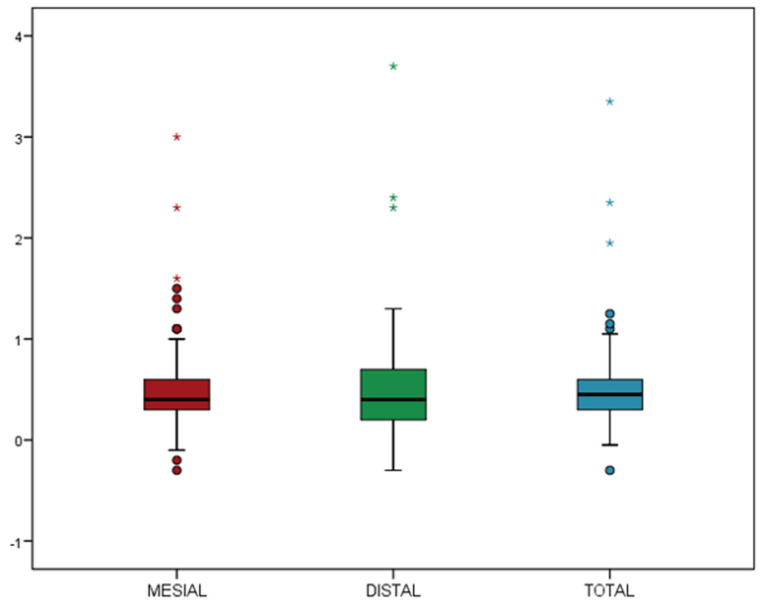
Box plot of the mean mesial, distal, and mean of both sites for MBL at 1-year follow-up. The box contains 50% of the cases, the median is the horizontal line that divides it. The upper and lower edges of the box correspond to the 1st and 3rd quartiles that represent 25% and 75% of the sampling, respectively. Whiskers in the box plots extend to the acceptable range of values (above which are the outliers (circles) and the extreme (asterisks) cases, respectively).

The MBL was calculated on the mesial and distal sides; it was then averaged for each implant. The mean MBL on the mesial and distal sides was 0.51 ± 0.44 mm and 0.52 ± 0.50 mm, respectively; the averaged MBL was 0.51 ± 0.44 mm, the median was 0.45 mm (Figure 2). 

**Figure 3:**
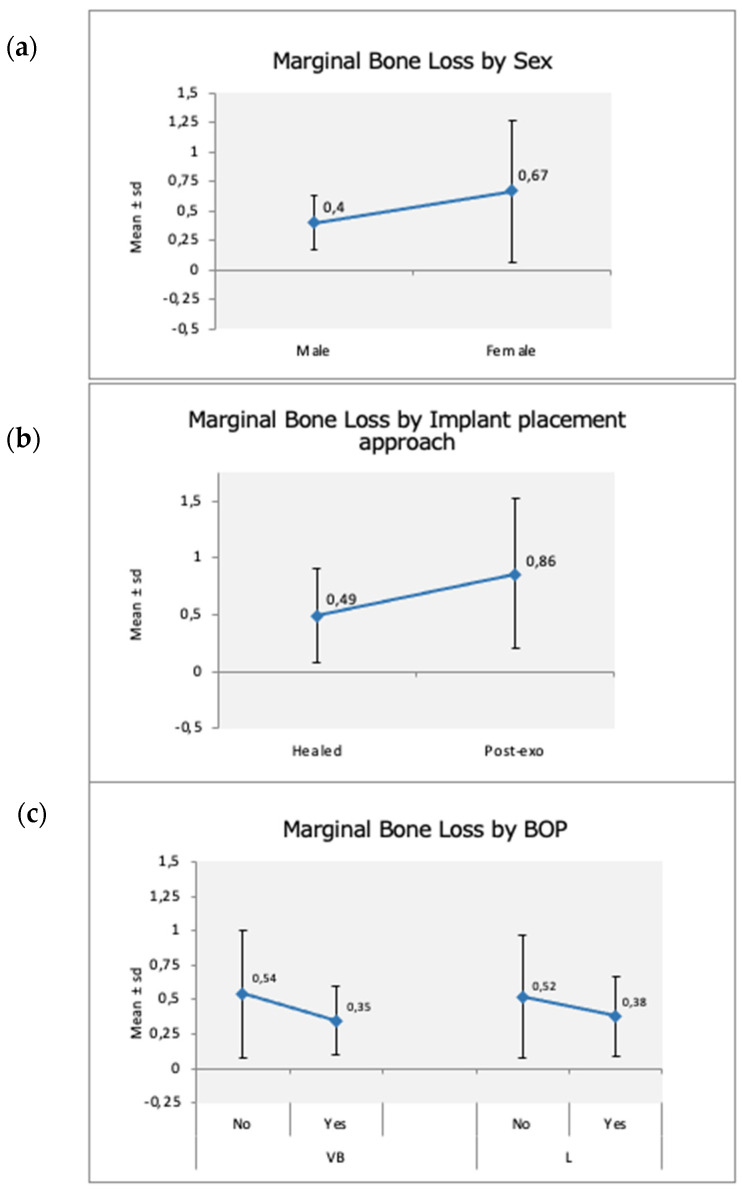
Significant clinical and patient-level parameters associated with MBL at 1-year follow-up. (**a**) MBL vs. gender; (**b**) MBL vs. timing of implant placement (healed ridge vs. post-extraction site); and (**c**) MBL vs. BoP.

The success rate was 96.7%. MBL was differently affected by the covariates of both the patient and the implant. Table 4 depicts the significant patient-level variables associated with MBL. Only gender was found to significantly affect the MBL at the 1-year follow-up; in female patients, the mean MBL was 0.67 ± 0.60 mm vs. 0.40 ± 0.23 mm in males. The mean 0.27 mm difference was statistically significant (*p* = 0.020) (Figure 3a). 

Among the other parameters, only the time of implantation, immediate or delayed, could be considered a relevant parameter (*p* < 0.10) (Table 4); immediate implants displayed a mean MBL of 0.86 ± 0.66 mm compared to 0.49 ± 0.42 mm for implants placed in healed sites (*p* = 0.095) (Table 3) (Figure 3b).

Table 5 shows how the clinical parameters related to the MBL. The buccal BoP measured at the 1-year follow-up was a good predictor of proximal MBL (*p* = 0.03). Figure 3c depicts the increase of MBL in presence of BoP at the buccal (*p* = 0.030) and lingual (*p* = 0.250) sides; however, only the vestibular side of the implant reached statistical significance. 

Lastly, history of periodontal disease (*p* = 0.428), smoking (*p* = 0.430), thickness of the gingiva (*p* = 0.399), soft tissue phenotype (*p* = 0.689), insertion torque (*p* = 0.232), implant-abutment disconnection with the MUA vs. disconnection with the Ti-base (*p* = 0.175), abutment height (*p* = 0.159), and all prosthetic variables did not significantly affect the MBL (Table 4 and Table 5). 

## 4. Discussion

Implant therapy is an adequate treatment for restoring dental function, aesthetics, and harmonization in the long term due to its high survival rate [1,2]. Most of the data published in the literature have been issued from either private clinical settings or universities by experimented teams with the aim to evaluate the changes over time of the health of the peri-implant tissues [1,16,17,18]. Those data depict the optimal results that can be expected from implant treatment, but have little connection with the real world of implant therapy in which a large number of implants are placed by poorly trained and poorly experimented surgeons. Of the several millions of implants placed each year in patients worldwide, most of them are inserted by practitioners for whom implant placement is not a daily activity [2]. In a retrospective study comparing experienced and non-experienced surgeons, Preiskel and Tsolka et al. [19] showed that experience had a major impact on the probability of implant failure [19]. More recently, Sendyk et al. [20] concluded that implant failure was significantly affected by the experience gained by the surgeon and the number of placed implants; a cut-off threshold of 50 implants was even identified [20].

The present data of this prospective clinical study stem from the daily treatment of a university setting with a large flow of patients that are treated by young 24- to 27-year-old inexperienced post-graduate students. Implant planning was performed under the supervision of a clinical instructor; however, the students did not have the learning curve that qualifies a surgeon as an expert. The inclusion criteria were not strict but large; they encompassed all sites, anterior in the esthetic area or in the posterior, healed or post-extractive, requiring or not vertical or horizontal bone regeneration with a resorbable membrane or sinus lifting. These render the current short-term results more representative of the genuine world of dental implantology treatment.

The success and survival rates of the 120 V3 implants were 97.5% and 96.7%, respectively; they were similar to the ones obtained by experimented university teams with the same implant [3,4,6,8,12]. They are also comparable to the survival features reported otherwise in the literature with other implant systems [16,17,18,21]. In a previous study published by our Department, the same inexperienced students placed and rehabilitated patients with the C1 implant, which displays a cylindrical collar and a rounded apex (MIS, Bar Lev Industrial Park, Israel), and the survival and success rates at 1-year were 96.15% and 94.62%, respectively [2]; those data were also similar to the ones obtained by experienced periodontists [22]. Our two studies with distinct implants showed that the lack of experience of the surgeons was not contributing to implant failure [2,22]. This is in contrast with the previously quoted studies [19,20]; the reason for that is unknown, but it might be that the tapered implant shape [23,24] and capacity to achieve satisfactory primary stability make these implants more forgiving than others in untried hands [23,24]. Noteworthily, the C1 and V3 implants are both provided with a disposable final drill. This means that final drilling is always performed with a clean and sharp tool; failure of osseointegration because of bone overheating due to worn-out drills can therefore be ruled out.

The triangular shape of the neck reduces the surface of the collar that comes in full contact with the cortical bone. By design and according to implant diameter, only 27–29% of the perimeter of the implant neck contacts the adjacent supporting bone [12]; this given warrants demonstration that primary stability is not jeopardized, especially in poor bone quality [12]. The mean IT was 37.1 Ncm, above the minimum requested IT of 32 Ncm that enables considering an immediate loading protocol [25]. In type 3 bone, the mean IT was 33.7 Ncm; in that bone quality, the literature reports ITs varying from 10 to 30 Ncm [26,27]. The mean and median IT in the posterior maxilla was 39.0 ± 10.4 and 40 Ncm, respectively; these numbers are in the same order of the ones, mean and median IT of 43.5 and 45 Ncm, provided by Eshkol-Yogev et al. [12] for the same implants placed in the same area. For comparison, the mean IT of Ti-Unite Branemark III implants placed in the posterior maxilla was 22 Ncm [27]. Noteworthy, in type 4 bone, the manufacturer recommends underdrilling the osteotomy by avoiding the final drill; the statistically significant difference measured between bone type 2 and 3 sites suggests that this advice should be carefully respected. To determine how much time is required until the gaps are filled with mineralized tissue, only three-dimensional assessments of the bone morphology in combination with bone cores obtained from patients will provide answers to the clinicians [5]. 

Steiner et al. [9] compared the IT features of three implant types; for the V3 implant, they described a steady increase of the IT followed by a relative decrease when the most coronal part of the neck passed the cortical layer. Indeed, the inexperienced surgeons that have been placing the C1 [2] and the V3 implants noticed a difference in the torque conduct close to the final seating [2]. For the C1, the sensation during implant placement in the osteotomy was a steady torque increase until reaching final seating; the C1 implant with its non-cutting round apex could not move beyond the prepared osteotomy, and the sensation was that of a robust immobilization and satisfactory primary stability. Insertion of the V3 in the prepared bony bed provided a distinct feeling. First, the IT increased steadily, like the C1, until the neck had fully engaged the alveolar ridge; upon final seating, the V3 implant with its cutting flat apex was able to move a little beyond the osteotomy shape. This vertical move decreased the compressive cortical support exerted at the contacting areas of the implant neck, and the feeling was that of a minor torque decrease instead of a stiff sensation. The surgeon placing the V3 in a healed ridge should be aware of these features: (1) final seating is not obtained when a firm immobilization is achieved, but when the planned neck position is reached, (2) in case the drilled osteotomy is too short to accommodate the implant length, it is still possible to seat the implant slightly deeper into the prepared osteotomy and reach the planned position of the implant neck.

The MBL at the 1-year control was 0.51 ± 0.44 mm; it was similar to the MBL reported by the experimented clinicians, 0.50 ± 0.40 mm by Eshkol-Yogev et al. [12] 3 months after placement and 10 disconnections of the implant-healing abutment junction, 0.22 ± 0.3 mm measured 1 year after loading by Li Manni et al. [6], and between 0.43 ± 0.37 mm for sites with thick gingiva to 1.25 ± 0.80 mm for sites with thin gingiva by Linkevicius et al. [8] 1-year after placement. Interestingly, the MBL measured in this study is similar to the MBL obtained in our previous study with the C1 implant [2]. Both implants provide a tight internal conical connection; for untrained prosthetic teams, this type of connection is somewhat more delicate to manage than an internal hex connection which provides a stopper to the abutment. The reason is that a tighter torque delivered to the abutment moves it slightly more apically.

The only parameter to significantly affect the MBL was gender, in which women exhibited a higher MBL than men, 0.67 ± 0.60 mm vs. 0.40 ± 0.23 mm in males (*p* = 0.020). Kolte et al. [28] also found that gender affected the MBL in their 7-year follow-up of posterior implants; however, a meta-analysis addressing this issue assessed that the literature was not conclusive with this regard [28]. The other covariate that showed a relevant association (*p* = 0.095) was timing of implant placement; the bone loss of the implants rehabilitating healed sites and post-extraction sockets was 0.49 ± 0.42 mm and 0.86 ± 0.66 mm, respectively. These data are in line with the literature [2,21] that denied a significant impact on the timing of implant placement in the long term [21].

Disconnection of the IAJ did not affect the MBL. At the MUA sites without disconnection, the MBL was 0.46 ± 0.77 mm vs. 0.54 ± 0.47 mm (*p* = 0.17) for the sites that underwent 3–5 disconnections, respectively. Some authors have recently documented a similar finding at the 1-year control [4], while at the 3-year control, others found an additional bone loss of 0.37–0.43 mm when disconnections have occurred, which was not considered clinically relevant [29,30,31]. Similarly, influence of the covariate ‘thickness of the gingiva’ failed to govern the MBL, a factor that has been claimed to be of significance for crestal bone loss [8,32,33,34,35]. 

A possible limitation of this study is that a clinical instructor helped the students with implant planning and supervised the surgery and prosthetic steps; this might explain the present positive outcome, which contrasts with other studies that reported that the experience of the surgeon is a covariate that affects the failure rate [19,20]. However, the fact that implants have been placed and rehabilitated by 24 distinct untrained individuals with a variety of natural skills may smooth down this bias.

## 5. Conclusions

Within the limitations of this prospective cohort study, the authors conclude that:

1. The survival and success rates of the V3 triangular-neck implants placed and by inexperienced post-graduate students at 1-year follow-up were 97.5% and 96.7%, respectively.
2. No contributing factors were identified regarding implant failure; however, a relevant association was found involving patients with smoking habits, where implant failure was five times higher (OR = 5.31, *p* = 0.09) in smokers.
3. The mean MBL was 0.51 ± 0.44 mm, and the inexperience of the rehabilitation team did not contribute to additional bone loss.
4. Gender was the single covariate to significantly impact the MBL.
5. Timing of implant placement, delayed vs. immediate, displayed a tendency to affect it, although not significantly.
6. BoP at the buccal sites of the implants was the only predictive factor of bone loss.

## Figures and Tables

**Table 1:** Variables recorded in this study.

Demographic Variables	Implant Variables	Surgical Variables	Prosthetic Variables
Age	Diameter	Corono-apical implant depth	Screw-retained
Gender	Length	Bone/sinus grafting	Cemented
Smoking	Local site	Healing protocol	Crown-implant ratio
Periodontal disease	Jaw	Insertion torque	
Controlled diabetes	Abutment height		
Oral hygiene	Soft tissue thickness		
Bone quality	Phenotype		
	Probing depth		
	Keratinized mucosa		
	Bleeding on probing		

**Table 2:** (**a**) Insertion torque according to implant diameter given in Ncm. (**b**) Insertion torque according to implant length given in Ncm.

**(a)**					
	**Ø 3.3 mm**	**Ø 3.9 mm**	**Ø 4.3 mm**	**Ø 5 mm**	***p***
	29.50%	28.80%	26.60%	29.00%	
Mean	40.0 ± 6.1	37.3 ± 12.2	36.40 ± 10.0	37.5 ± 10.1	>0.05
Median	40	40	35	37.5	
**(b)**					
	**8 mm**	**10 mm**	**11.5 mm**		**13 mm**
Mean	36.1 ± 11.8	40.5 ± 9.6	34.8 ± 11.1		35.0 ± 7.8
Median	40	40	35		35

**Table 3:** Probability of implant failure according to the independent analyzed variables.

Implant Failure	Category *	OR	CI 95%	*p*-Value
**Sex**	Male (*n* = 25)	1	-	-
	Female (*n* = 22)	2.78	0.26–29.3	0.396
**Smoking habits**	No (*n* = 11)	1	-	-
	Yes (*n* = 36)	5.31	0.48–58.3	**0.172**
**Controlled Diabetes**	No (*n* = 40)	-	-	1.000
	Yes (*n* = 7)	-	-	
**History of Periodontitis**	No (*n* = 26)	-	-	0.244
	Yes (*n* = 21)	-	-	
**Sector**	Anterior (*n* = 41)	1	-	-
	Posterior (*n* = 79)	1.04	0.09–11.8	0.975
**Jaw**	Maxilla (*n* = 66)	1	-	-
	Mandible (*n* = 54)	2.50	0.21–29.7	0.468
**Implant diameter (mm)**		0.91	0.58–1.41	0.659
**Implant length (mm)**		0.94	0.69–1.29	0.691
**Surgical protocol**	1 stage (*n* = 46)	-	-	0.522
	2 stages (*n* = 73)	-	-	

* *p* < 0.05; ** *p* < 0.01; *** *p* < 0.001. * Sample: patient level *n* = 47; implant level *n* = 120. Results for the Wald Chi-squared test of a simple binary logistic regression model (for generalized estimation equations) association between failure and different variables at both patient and implant level. Relevant association and tendency with MBL are in bold.

**Table 4:** Association between total MBL vs. independent covariates for patient profile, surgical protocol, and implant characteristics.

MBL vs. Independent Variables	Category	Beta	CI 95%	*p*-Value
**Sex**	Male (0.40 ± 0.23)	0		
	Female (0.67 ± 0.60)	0.27	0.04–0.48	0.020 *
**Smoking habits**	No (0.48 ± 0.36)	0		
	Yes (0.60 ± 0.61)	0.12	−0.17–0.40	0.430
**Controlled Diabetes**	No (0.49 ± 0.43)	0		
	Yes (0.62 ± 0.53)	0.12	−0.18–0.42	0.424
**History of Periodontitis**	No (0.55 ± 0.56)	0		
	Yes (0.47 ± 0.26)	−0.08	−0.27–0.12	0.428
**Sector of the jaw**	Anterior (0.57 ± 0.61)	0		
	Posterior (0.48 ± 0.33)	−0.09	−0.28–0.09	0.332
**Jaw**	Maxilla (0.57 ± 0.55)	0		
	Mandible (0.44 ± 0.25)	−0.13	−0.31–0.06	0.185
**Implant diameter (mm)**	(0.51 ± 0.44)	−0.05	−0.14–0.04	0.294
**Implant length (mm)**	(0.51 ± 0.44)	0.03	−0.01–0.06	0.151
**Surgical protocol**	1 stage (0.51 ± 0.40)	0		
	2 stages (0.52 ± 0.47)	0.01	−0.17–0.18	0.960
**Implant site**	Healed ridge (delayed) (0.49 ± 0.42)	0		
	post-exo (immediate) (0.86 ± 0.66)	0.37	−0.07–0.81	**0.095**
**Bone grafting**	No (0.49 ± 0.46)	0		
	Yes (0.63 ± 0.35)	0.13	−0.08–0.35	0.212
**Implant depth at placement**	(0.51 ± 0.44)	−0.19	−0.50–0.12	0.227
**Torque (Ncm)**	(0.48 ± 0.35)	0.00	−0.01–0.01	0.232

* *p* < 0.05; ** *p* < 0.01; *** *p* < 0.001. * Mean MBL in mm. Wald Chi-squared test results of a general linear regression model. Statistically significant correlations with MBL are in bold.

**Table 5:** Association between MBL and other clinical parameters.

Parameters	Category *	Beta	CI 95%	*p*-Value
**BoP (Buccal)**	No (0.54 ± 0.46)	0		
	Yes (0.35 ± 0.25)	−0.19	−0.36–−0.02	0.030 *
**BoP (lingual/palatal)**	No (0.52 ± 0.45)	0		
	Yes (0.38 ± 0.29)	−0.14	−0.38–0.10	0.250
**PD total**	(0.51 ± 0.44)	−0.03	−0.15–0.10	0.682
**Plaque (Buccal)**	No (0.51 ± 0.43)	0		
	Yes (0.5 ± 0.55)	0.04	−0.27–0.36	0.775
**Plaque (lingual/palatal)**	No (0.51 ± 0.43)	0		
	Yes (0.50 ± 0.55)	−0.01	−0.31–0.29	0.948
**KT**		−0.03	−0.12–0.05	0.399
**KT groups**	<2 mm (0.56 ± 0.44)	0		
	>2 mm (0.50 ± 0.45)	−0.06	−0.27–0.15	0.594
**Ti-base**	No (0.47 ± 0.34)	0		
	Yes (0.54 ± 0.50)	0.07	−0.05–0.19	0.240
**Multi-unit**	No (0.54 ± 0.47)	0		
	Yes (0.46 ± 0.37)	−0.09	−0.21–0.04	0.175
**Soft tissue phenotype**	Thin (0.51 ± 0.49)	0		
	Thick (0.55 ± 0.44)	0.01	−0.25–0.26	0.965
**Gingival Thickness**	(0.80 ± 1.04)	−0.04	−0.25–0.16	0.689
**Bone Quality**	(0.51 ± 0.44)	0.16	−0.05–0.37	0.130
**Papilla index (mesial)**	(0.52 ± 0.46)			0.908
	0 (0.51 ± 0.38)	0		
	1 (0.52 ± 0.51)	0.01	−0.36–0.37	0.973
	2 (0.51 ± 0.39)	−0.01	−0.38–0.36	0.964
**Papilla index (distal)**	(0.52 ± 0.46)			0.684
	0 (0.54 ± 0.60)	0		
	1 (0.52 ± 0.40)	−0.02	−0.28–0.24	0.894
	2 (0.45 ± 0.28)	−0.08	−0.37–0.20	0.572

* *p* < 0.05; ** *p* < 0.01; *** *p* < 0.001. * Mean MBL in mm. Wald Chi-squared test results of a general linear regression model. Statistically significant correlations with MBL are in bold.

## Data Availability

Not applicable.
